# The short chain fatty acid receptor GPR43 regulates inflammatory signals in adipose tissue M2-type macrophages

**DOI:** 10.1371/journal.pone.0179696

**Published:** 2017-07-10

**Authors:** Akira Nakajima, Akiho Nakatani, Sae Hasegawa, Junichiro Irie, Kentaro Ozawa, Gozoh Tsujimoto, Takayoshi Suganami, Hiroshi Itoh, Ikuo Kimura

**Affiliations:** 1 Department of Applied Biological Science, Graduate School of Agriculture, Tokyo University of Agriculture and Technology, Fuchu-shi, Tokyo, Japan; 2 Department of Internal Medicine, School of Medicine, Keio University, Shinjuku-ku, Tokyo, Japan; 3 Department of Pharmacology, Nara Medical University School of Medicine, Nara, Japan; 4 Department of Genomic Drug Discovery Science, Graduate School of Pharmaceutical Sciences, Kyoto University, Shimoadachi-cho, Sakyo-ku, Kyoto, Japan; 5 Department of Molecular Medicine and Metabolism, Research Institute of Environmental Medicine, Nagoya University, Furo-cho, Chikusa-ku, Nagoya, Japan; Tokyo University of Agriculture, JAPAN

## Abstract

The regulation of inflammatory responses within adipose tissue by various types of immune cells is closely related to tissue homeostasis and progression of metabolic disorders such as obesity and type 2 diabetes. G-protein-coupled receptor 43 (GPR43), which is activated by short-chain fatty acids (SCFAs), is known to be most abundantly expressed in white adipose tissue and to modulate metabolic processes. Although GPR43 is also expressed in a wide variety of immune cells, whether and how GPR43 in adipose tissue immune cells regulates the inflammatory responses and metabolic homeostasis remains unknown. In this study, we investigated the role of GPR43 in adipose tissue macrophages by using *Gpr43*-deficient mice and transgenic mice with adipose-tissue-specific overexpression of GPR43. We found that GPR43 activation by SCFA resulted in induction of the pro-inflammatory cytokine tumor necrosis factor-α (TNF-α) in anti-inflammatory M2-type macrophages within adipose tissue. By contrast, this effect was not noted in inflammatory M1-type macrophages, suggesting that GPR43 plays distinct functions depending on macrophage types. Local TNF-α signaling derived from steady-state adipose tissue is associated with proper tissue remodeling as well as suppression of fat accumulation. Thus, GPR43-involving mechanism that we have identified supports maintenance of adipose tissue homeostasis and increase in metabolic activity. This newly identified facet of GPR43 in macrophages may have clinical implications for immune-metabolism related episodes.

## Introduction

Metabolic disorders such as obesity and type 2 diabetes have become a global epidemic, with incidence rates of over 30% in most Western countries[1]. The obesity epidemic is a result of imbalances in whole-body energy regulation[2]. White adipose tissue (WAT), which is composed of adipocytes and the stromal vascular fraction (SVF; a heterogeneous mixture of mesenchymal, endothelial, and various immune cells), is a central metabolic organ involved in the regulation of energy homeostasis[3,4]. Current research efforts have indicated that various cell types of the immune system within the WAT play a significant role in the regulation of metabolic homeostasis and that disruption of this immune system is closely associated with obesity and type 2 diabetes[5–8]. In the steady state, various anti-inflammatory immune cells such as M2-type macrophages, eosinophils, group 2 innate lymphoid cells (ILC2s), invariant natural killer T (iNKT) cells, T helper type 2 (Th2) cells and regulatory T (Treg) cells are engaged in homeostasis in the WAT[9–14]. However, during progression of obesity, type 2 immune cells in the WAT become dysregulated and accumulated inflammatory responses in the WAT play a causal role in the deleterious effects on metabolism[15,16]. Among the immune cells in the WAT, macrophages are the most abundant in the leukocyte population and generally contribute to inflammation-mediated insulin resistance[17]. Therefore, understanding the inflammatory responses involving macrophages within adipose tissue is clearly of clinical importance.

G-protein-coupled receptor 43 (GPR43), also called free fatty acid receptor 2 (FFAR2), binds short-chain fatty acids (SCFAs; acetate, propionate and butyrate) produced by the microbial fermentation of carbohydrates. Emerging research has suggested that GPR43, which is expressed in adipose tissue, pancreatic islets, and gastrointestinal tract, is involved in modulating metabolic processes[18–20]. For instance, GPR43-deficient (*Gpr43*^*-/-*^) mice develop obesity when they are on high fat and normal chow diets, and GPR43 activation increases energy expenditure and preferentially enables fat consumption via inhibition of insulin signaling in the WAT[18]. Moreover, GPR43 is also expressed on a wide variety of immune cells, including neutrophils, macrophages, dendritic cells, mast cells and lymphocytes, and is involved in regulating inflammatory responses[21–25].

The role of GPR43 in the regulation of inflammatory responses remains obscure or even controversial. It has been reported that GPR43 signaling reduces inflammatory mediator production and affects inflammatory leukocyte migrations[22]. However, evidence has also been obtained that GPR43 stimulation causes inflammasome-dependent acute inflammatory responses in macrophages, leading to neutrophil recruitment[24]. In addition, the extent to which GPR43 contributes to inflammatory responses within the WAT has not been rigorously examined.

In this study, we investigated the role of GPR43 in the adipose tissue macrophages by using *Gpr43*^*-/-*^ mice and transgenic mice with adipose-tissue specific overexpression of GPR43. We found that GPR43, which is expressed in anti-inflammatory M2-type macrophages within the WAT, critically contributed to the induction of tumor necrosis factor-α (TNF-α), depending on SCFA stimulation. However, this effect of GPR43 was not seen in the other types of macrophages such as inflammatory M1-type macrophages. Inflammatory signaling in steady-state adipose tissue has been shown to be necessary for tissue homeostasis and for enhancing insulin sensitivity[26]. Thus, our results underscore the role of GPR43 as an intervening factor between immune and metabolic systems for modulation of homeostasis in adipose tissue.

## Results

### *Gpr43* is expressed in the adipose tissue SVF as well as in mature adipocytes

**Fig 1:**
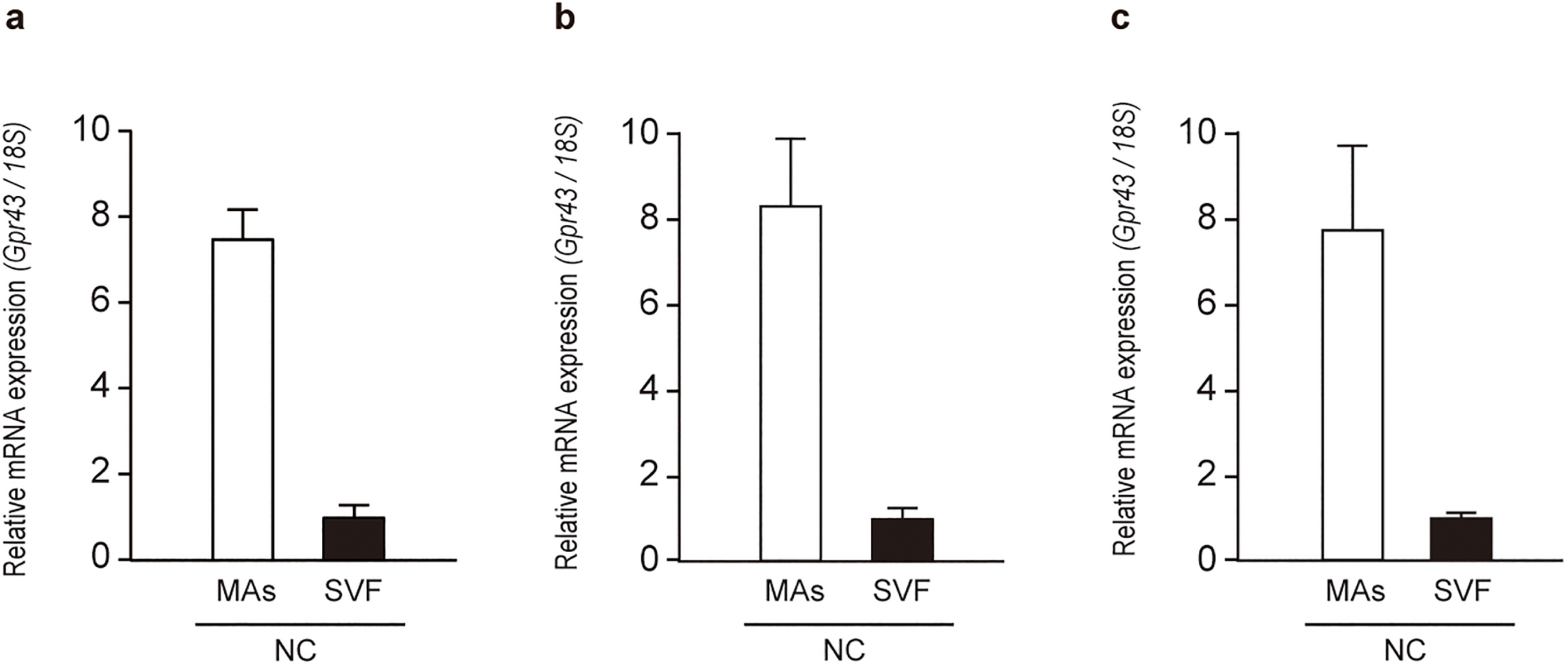
*Gpr43* is expressed in the adipose tissue SVF. Expression of *Gpr43* mRNA in the adipose tissue MAs and SVF of NC-fed mice by qRT-PCR (n = 3). Mesenteric adipose tissue (a), epididymal adipose tissue (b) and subcutaneous adipose tissue (c). *18S* mRNA expression was used as an internal control. All data are presented as mean ± S.E.M. MAs: mature adipocyte, SVF: stromal vascular fraction.

To investigate whether GPR43 plays some functions in immune cells within the WAT, we initially divided the mesenteric adipose tissue of normal chow (NC)-fed mice into mature adipocytes (MAs) and the SVF (S1A Fig), and analyzed *Gpr43* mRNA expression in each fraction by quantitative reverse-transcription PCR (qRT-PCR). Although high *Gpr43* mRNA levels were noted in MAs as previously reported[18], we could also detect *Gpr43* mRNA expression in the SVF, albeit the expression levels were lower than those in MAs (Fig 1A). Similar observations were made in epididymal adipose tissue and subcutaneous adipose tissue (Fig 1B and 1C and S1B and S1C Fig), and *Gpr43* expression in the SVF was also observed in high-fat diet (HFD)-fed mice (S1D–S1F Fig). These results prompted us to investigate whether GPR43 plays some kind of roles in adipose tissue immune cells.

### Adipose-specific *Gpr43* transgenic mice are lean and enhance insulin sensitivity

**Fig 2:**
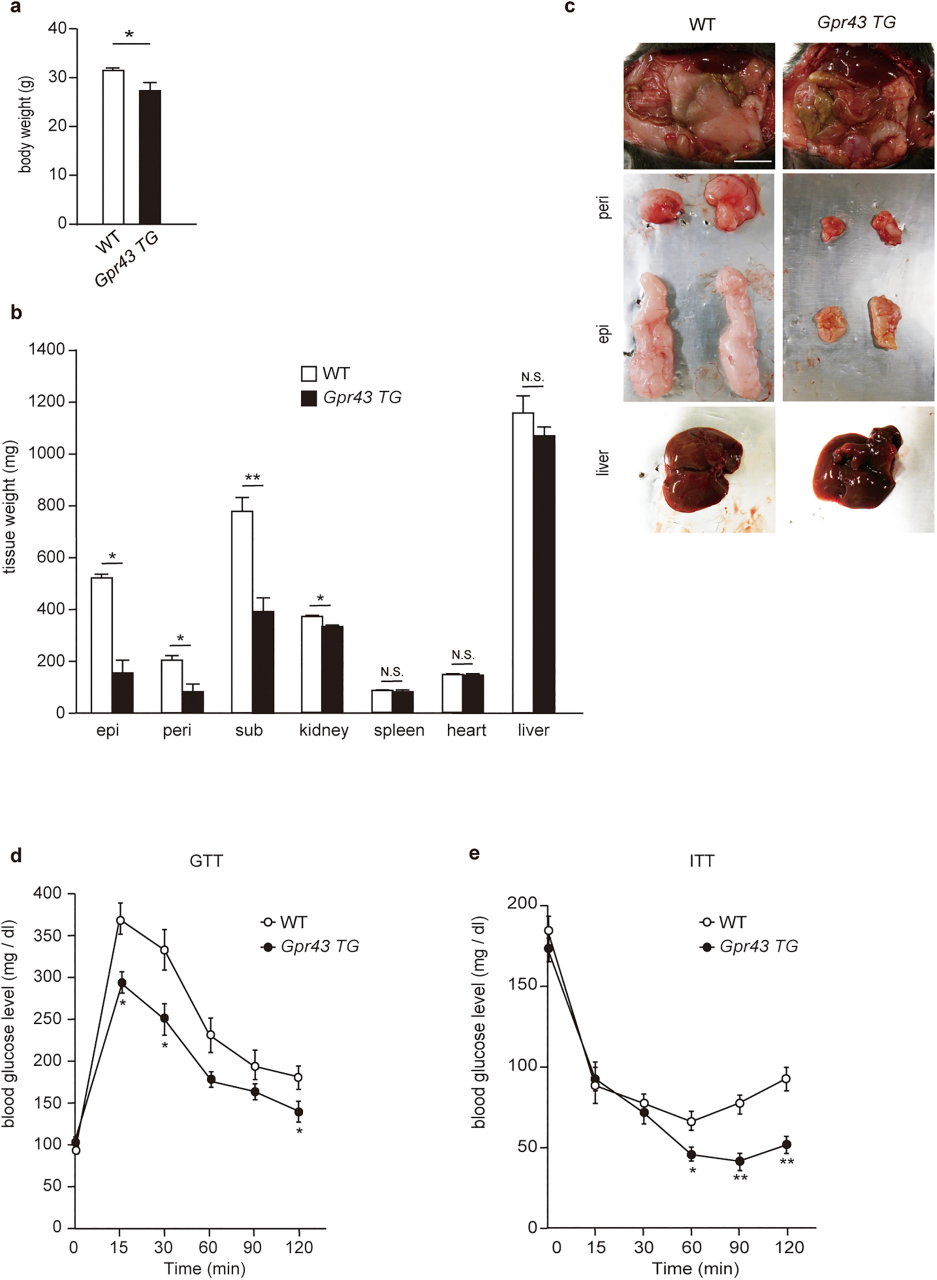
Adipose-specific *Gpr43* transgenic mice (*aP2-Gpr43TG* mice) are lean and enhance insulin sensitivity. Body weights (a), tissue weights (b), and macroscopic appearance (c) of control WT and *aP2-Gpr43TG* mice fed an NC (n = 6−8). Scale bar: 10 mm. GTT (d) and ITT (e) in control WT and *aP2-Gpr43TG* mice fed an NC (n = 6−8). Mice were analyzed at 16 weeks of age. All data are presented as mean ± S.E.M. *p < 0.05, **p < 0.01, N.S.: not significant. epi: epididymal adipose tissue, peri: perirenal adipose tissue, sub: subcutaneous adipose tissue.

To determine the role of GPR43 in adipose tissue, we used adipocyte fatty-acid binding protein (aP2; expressed in adipocytes and macrophages) promoter-driven adipose-specific human Gpr43 transgenic mice (*aP2-Gpr43TG*)[18]. In agreement with our previous results[18], the body weights and the WAT weights of *aP2-Gpr43TG* mice were significantly lower than those of wild-type (WT) mice in the NC-fed condition. However, the weights of other tissues were not dramatically different between WT and *aP2-Gpr43TG* mice, indicating that *aP2-Gpr43TG* mice did not have ectopic fat deposition (i.e., deposition of triglycerides within cells of non-adipose tissue; Fig 2A–2C). Moreover, *aP2-Gpr43TG* mice showed beneficial metabolic effects as compared to those seen in age-matched WT mice, that is, smaller glucose excursions during glucose tolerance tests (GTT) and increased sensitivity during insulin tolerance tests (ITT) (Fig 2D–2E). We have previously shown that insulin-induced Akt phosphorylation in the WAT, but not the muscles or liver, was markedly suppressed in *aP2-Gpr43TG* mice in comparison to that in WT mice[18]. Therefore, these results suggest that GPR43 overexpression in adipose tissue suppressed fat accumulation by impairing insulin signaling selectively in the WAT and consequently enhanced insulin sensitivity.

### TNF-α is highly produced in adipose tissue of *aP2-Gpr43TG* mice

**Fig 3:**
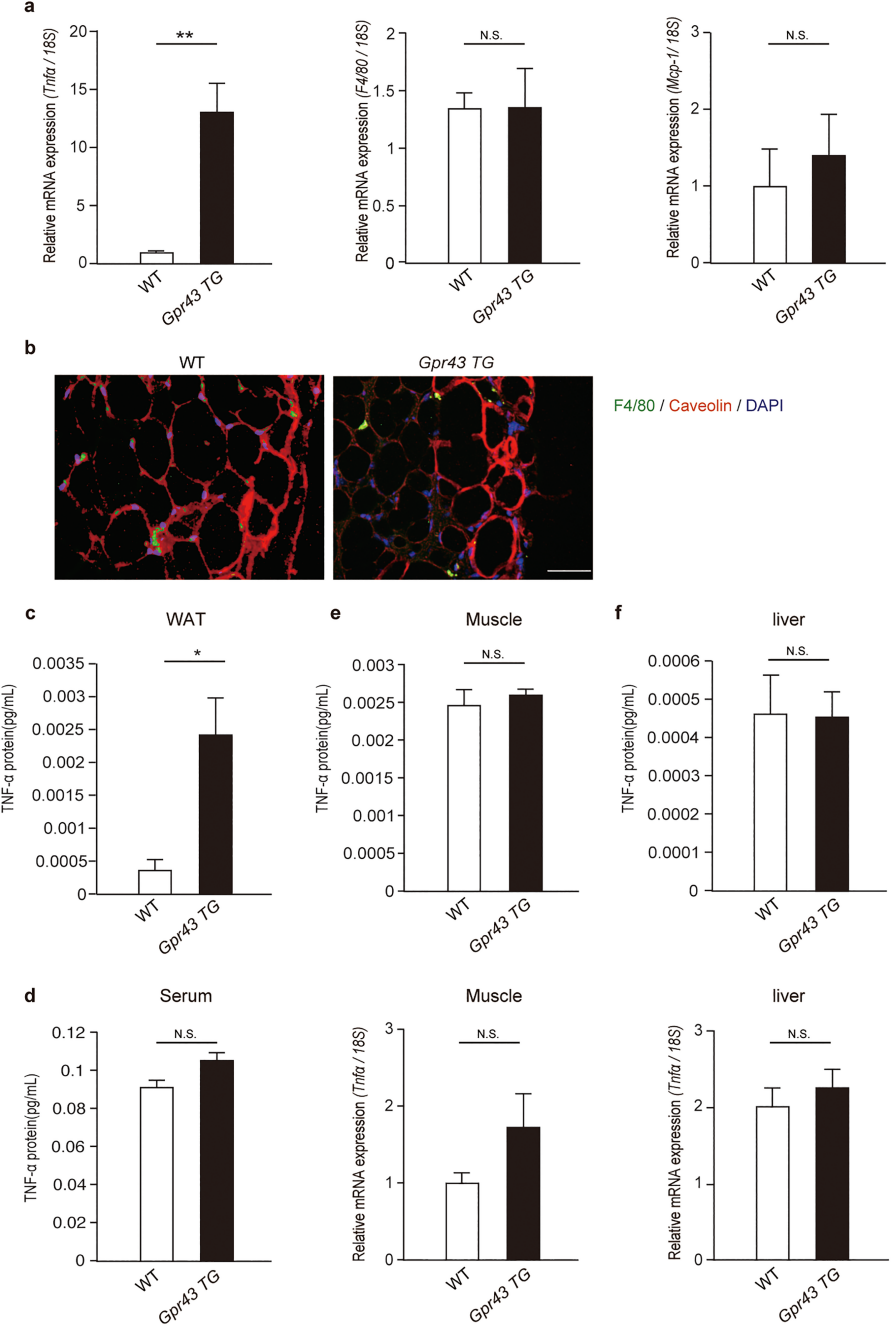
TNF-α is highly produced in adipose tissue of *aP2-Gpr43TG* mice. (a) *Tnfα*, *F4/80*, and *Mcp-1* mRNA expression in the epididymal adipose tissue of control WT and *aP2-Gpr43TG* mice fed an NC, measured using qRT-PCR (n = 6−8). (b) Epididymal adipose tissue stained with anti-F4/80 (green), anti-caveolin 1 (red) and 4’,6-diamidino-2-phenylindole (DAPI) (blue). Scale bar: 50 μm. (c) ELISA for TNF-α in the epididymal adipose tissue of control WT and *aP2-Gpr43TG* mice fed an NC (n = 6−8). (d) ELISA for TNF-α in the serum of control WT or *aP2-Gpr43TG* mice fed an NC (n = 6−8). (e) ELISA for TNF-α (upper) and *Tnfα* mRNA expression (lower) in the muscle (n = 6−8). (f) ELISA for TNF-α (upper) and *Tnfα* mRNA expression (lower) in the liver (n = 6−8). *18S* mRNA expression was used as an internal control. Mice were analyzed at 16 weeks of age. All data are presented as mean ± S.E.M. *p < 0.05, **p < 0.01, N.S.: not significant.

For better understanding of the role of GPR43 in the WAT, we examined the expression profiles of various genes in the epididymal adipose tissue of WT and *aP2-Gpr43TG* mice. Surprisingly, mRNA expression of the pro-inflammatory cytokine *Tnfα* was much higher in *aP2-Gpr43TG* mice than WT mice, even if *aP2-Gpr43TG* mice were leaner (Fig 3A). Moreover, an enzyme-linked immunosorbent assay (ELISA) revealed that a large amount of TNF-α protein was produced in the WAT of *aP2-Gpr43TG* mice (Fig 3C). Although macrophages are one of the main TNF-α-producing cell types, the mRNA expression of *F4/80* (macrophage marker) and *Mcp-1* (monocyte chemoattractant protein-1; one of the key chemokines that regulate migration and infiltration of monocytes/macrophages) did not differ between both types of adipose tissue (Fig 3A and 3B). In contrast, these distinctions were not seen in the entire body; in the plasma and in other tissues such as muscles and the liver, *Tnfα* mRNA and protein expression of TNF-α did not differ between WT and *aP2-Gpr43TG* mice (Fig 3D–3F). Collectively, these results suggest that a large amount of TNF-α was selectively produced in the WAT of *aP2-Gpr43TG* mice in comparison to WT mice.

### *Tnfα* mRNA expression is strongly induced in adipose tissue macrophages in *aP2-Gpr43TG* mice

**Fig 4:**
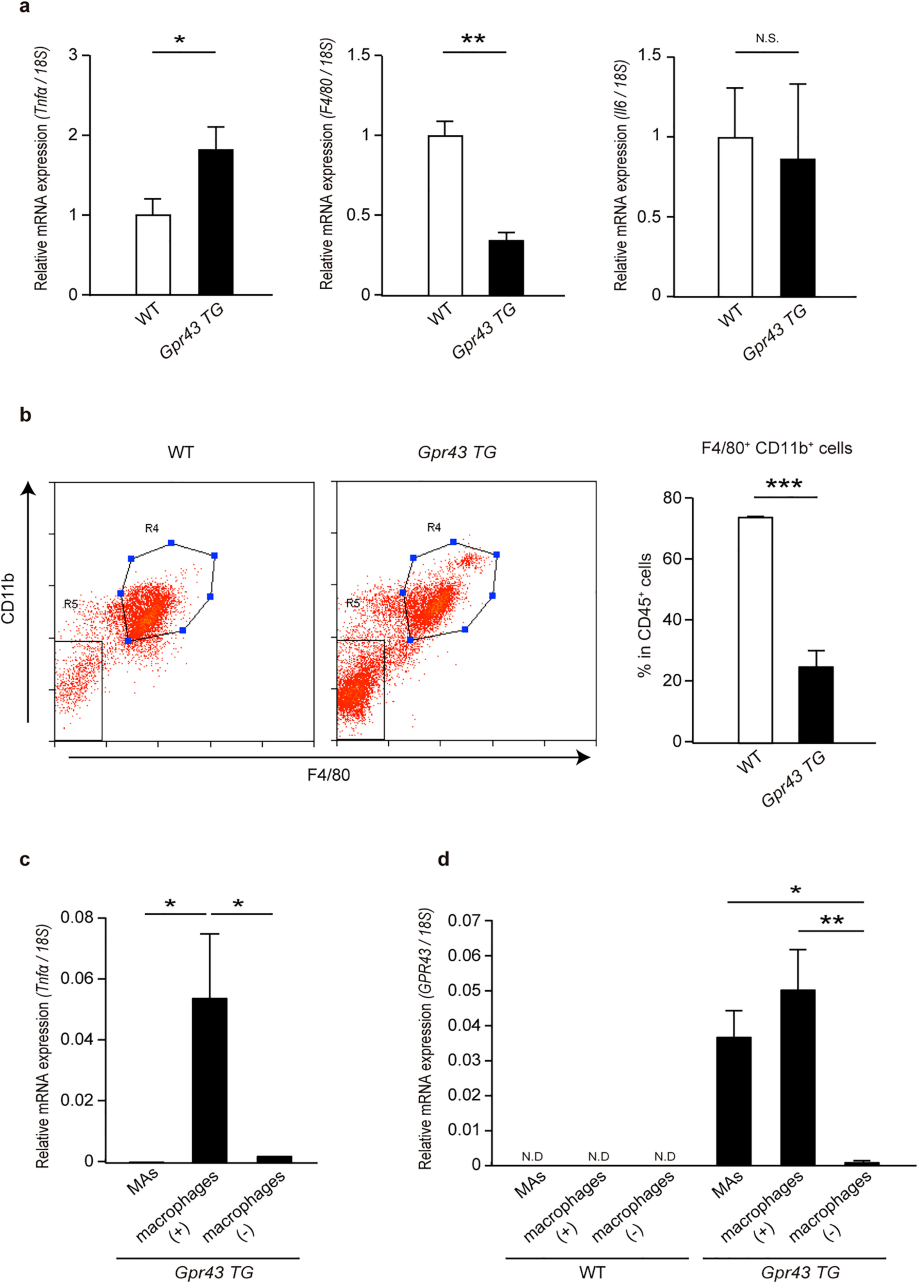
*Tnfα* mRNA is strongly induced in adipose tissue macrophages of *aP2-Gpr43TG* mice. (a) *Tnfα*, *F4/80*, and *Il6* mRNA expression in the epididymal adipose tissue SVF of control WT and *aP2-Gpr43TG* mice fed an NC measured using qRT-PCR (n = 3). (b) Representative flow cytometric plots (left) and bar graphs summarizing the data for CD11b^+^F4/80^+^ expression by live CD45^+^ cells (right) extracted from the epididymal adipose tissue of control WT and *aP2-Gpr43TG* mice (n = 3−4). R4: CD45^+^CD11b^+^F4/80^+^ cells, R5: CD45^+^CD11b^-^F4/80^-^ cells. (c) *Tnfα* mRNA expression in epididymal adipose tissue MAs, macrophage subsets, and non-macrophage subsets of *aP2-Gpr43TG* mice fed an NC, measured using qRT-PCR (n = 4). (d) *GPR43* mRNA expression in epididymal adipose tissue MAs, macrophage subsets, and non-macrophage subsets of control WT or *aP2-Gpr43TG* mice fed an NC, measured using qRT-PCR (n = 3−4). *18S* mRNA expression was used as an internal control. Mice were analyzed at 35–40 weeks of age. All data are presented as mean ± S.E.M. *p < 0.05, **p < 0.01, ***p < 0.001, N.S.: not significant.

Since TNF-α is produced from various types of immune cells such as macrophages, dendritic cells, CD4^+^T cells, and CD8^+^T cells, we examined whether the immune cells in the WAT of *aP2-Gpr43TG* mice were responsible for producing a large amount of TNF-α. To test this, we purified the SVF from the epididymal adipose tissue of WT and *aP2-Gpr43TG* mice and compared TNF-α gene induction. As expected, *Tnfα* mRNA expression was induced at a much higher level in the SVF of *aP2-Gpr43TG* mice than in that of WT mice, even if *F4/80* mRNA expression was much lower (Fig 4A); in contrast, no significant differences were seen in interleukin-6 (*Il6*) mRNA induction (Fig 4A). These observations raised the interesting issue of what cell types mainly produced TNF-α in the *aP2-Gpr43TG* mice.

To determine this, the epididymal adipose tissue from WT and *aP2-Gpr43TG* mice was divided into the MAs and SVF, and then the SVF was further sorted into macrophage (F4/80^+^CD11b^+^) subsets and non-macrophage (F4/80^-^CD11b^-^) subsets by using a flow cytometry approach (sorting schema and purity achieved are shown in S2 Fig). Although the frequency of macrophages within immune cells was much lower in *aP2-Gpr43TG* mice than in WT mice, robust *Tnfα* mRNA induction was observed in macrophages of *aP2-Gpr43TG* mice; it was barely detectable in non-macrophage subsets and MAs (Fig 4B and 4C). aP2 promoter-driven constructs are predominantly transcribed in adipocytes but their expression has also been reported in macrophages, although at much lower levels[27]; therefore, we examined human *GPR43* mRNA expression level in each fraction. The adipose tissue macrophages of *aP2-Gpr43TG* mice showed higher *GPR43* mRNA expression than non-macrophage immune cells (Fig 4D). As expected, although MAs showed high *GPR43* expression, *GPR43* was also abundantly expressed in macrophages; the expression level was equal to or higher than that in MAs, suggesting that the adipose tissue macrophages of *aP2-Gpr43TG* mice showed high *GPR43* expression and produced a large amount of TNF-α. These results raised the possibility that GPR43 could directly regulate the gene induction of TNF-α in macrophages.

### GPR43 is essential for inducing *Tnfα* mRNA expression in SCFA-stimulated M2-type macrophages but not M1-type macrophages *in vitro*

**Fig 5:**
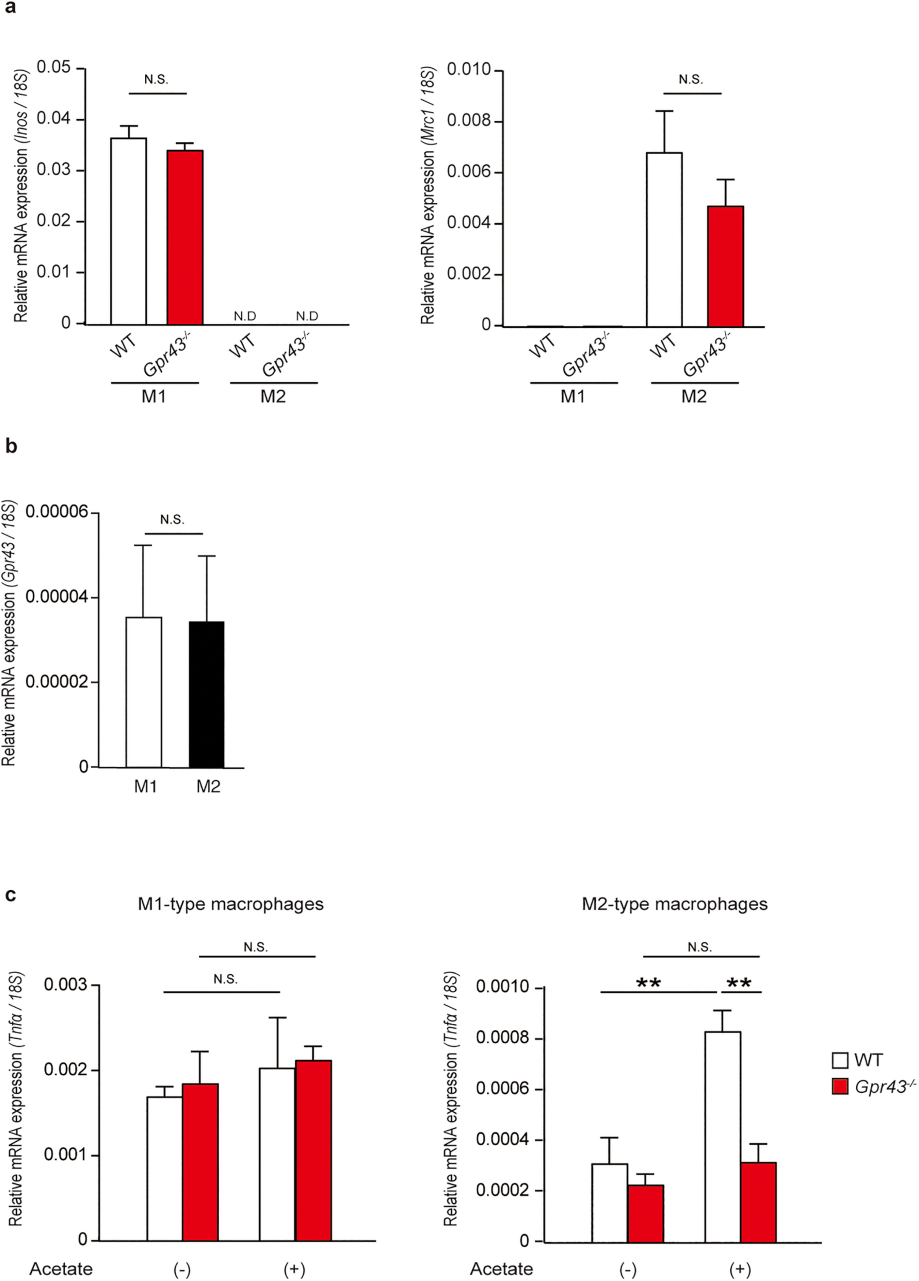
GPR43 is essential for inducing *Tnfα* mRNA expression in SCFA-stimulated M2-type macrophages but not in M1-type macrophages *in vitro*. (a) Quantitative RT-PCR analysis of *Inos* (left) and *Mrc1* (right) mRNA expression in WT and *Gpr43*^*-/-*^ M1 and M2 bone marrow derived macrophages (n = 3). *18S* mRNA expression was used as an internal control. (b) Quantitative RT-PCR analysis of *Gpr43* mRNA expression in WT M1 and M2 bone marrow derived macrophages (n = 3). *18S* mRNA expression was used as an internal control. (c) Quantitative RT-PCR analysis of *Tnfα* mRNA expression in WT and *Gpr43*^*-/-*^ M1 (left) and M2 (right) bone marrow derived macrophages stimulated for 7 h with acetate (10 mM) (n = 3). *18S* mRNA expression was used as an internal control. All data are presented as mean ± S.E.M. **p < 0.01, N.S.: not significant. Data are representative of three experiments (a-c).

Tissue macrophages respond to changes in the local environment by changing their polarization; the main populations of adipose tissue macrophages that reside in lean adipose tissue differ from those residing in obese adipose tissues. In the lean status, the predominant adipose tissue macrophage population is M2-type non-inflammatory macrophages, which express high levels of the mannose receptor (*Mrc1* encoding MR, also known as CD206), arginase-1 (*Arg1*), and CD301 and secrete anti-inflammatory cytokines including IL-10 and IL-1 receptor antagonists[28]. In contrast, in obesity, interferon (IFN)-gamma and lipopolysaccharide (LPS) drive the polarization of recruited monocytes toward inflammatory M1-type macrophages and produce a large amount of nitric oxide by expressing inducible nitric oxide synthase (*Inos*), TNF-α, IL-6, IL-1β, IL-12, and MCP-1[29]. Therefore, to investigate the functional roles of GPR43 in macrophages, we polarized bone marrow-derived macrophages from WT and *Gpr43*^*-/-*^ mice into M1- or M2-type macrophages and examined the inflammatory responses, depending on SCFA stimulation *in vitro*. We confirmed that robust *Inos* mRNA induction occurred in M1-polarized macrophages and that induction of *Mrc1* mRNA expression was not detectable, whereas the converse was observed in M2-polarized macrophages (Fig 5A). *Gpr43*^*-/-*^ cells showed no difference in polarization to M1- or M2-type macrophages in comparison to WT cells, and *Gpr43* mRNA expression did not differ between M1- and M2-type macrophages in WT cells (Fig 5A and 5B), indicating that GPR43 did not directly affect macrophages polarizations.

On stimulation with acetate, which is the most selective SCFA ligand for GPR43, induction of *Tnfα* mRNA expression was not observed in either WT or *Gpr43*^*-/-*^ M1-type macrophages. However, *Tnfα* mRNA expression levels increased after acetate stimulation in WT but not *Gpr43*^*-/-*^ M2-type macrophages (Fig 5C). These findings collectively suggest that SCFA-activated GPR43 contributed to *Tnfα* gene induction in M2-type macrophages, but not in M1-type macrophages.

Since GPR43 may modulate *Tnfα* gene expression via mitogen-activated protein kinase (MAPK) signaling[30,31], we further examined GPR43-mediated intracellular signaling in M2-type macrophages. Western blot analysis of cell lysates from WT and *Gpr43*^*-/-*^ M2-type macrophages revealed that extracellular signal-regulated kinase 1 (Erk1) was phosphorylated in WT macrophages after acetate stimulation, but this was strongly suppressed in *Gpr43*^*-/-*^ macrophages (S3A Fig). Thus, GPR43 activation by SCFA led to promotion of *Tnfα* gene induction in M2-type macrophages via MAPK signaling.

We examined *Tnfα* mRNA induction in thioglycollate-elicited peritoneal exudate cells (PECs) stimulated with acetate or propionate. In contrast to the findings for M2-type bone marrow-derived macrophages, *Tnfα* mRNA expression was suppressed in WT thioglycollate-elicited PECs, depending on SCFA stimulation; this was not observed in *Gpr43*^*-/-*^ thioglycollate-elicited PECs (S3B Fig). Thioglycollate-induced PECs have been reported to have populations similar to those of PECs stimulated with LPS[32]; apparently SCFA-activated GPR43 suppressed the inflammatory responses in M1-type inflammatory macrophages (S3B Fig). As such, GPR43-mediated inflammatory responses may need to be fine-tuned so as to evoke it on the one hand and suppress it on the other.

### *Tnfα* mRNA expression is induced by adipose tissue M2-type macrophages, depending on GPR43 and SCFA stimulation

**Fig 6:**
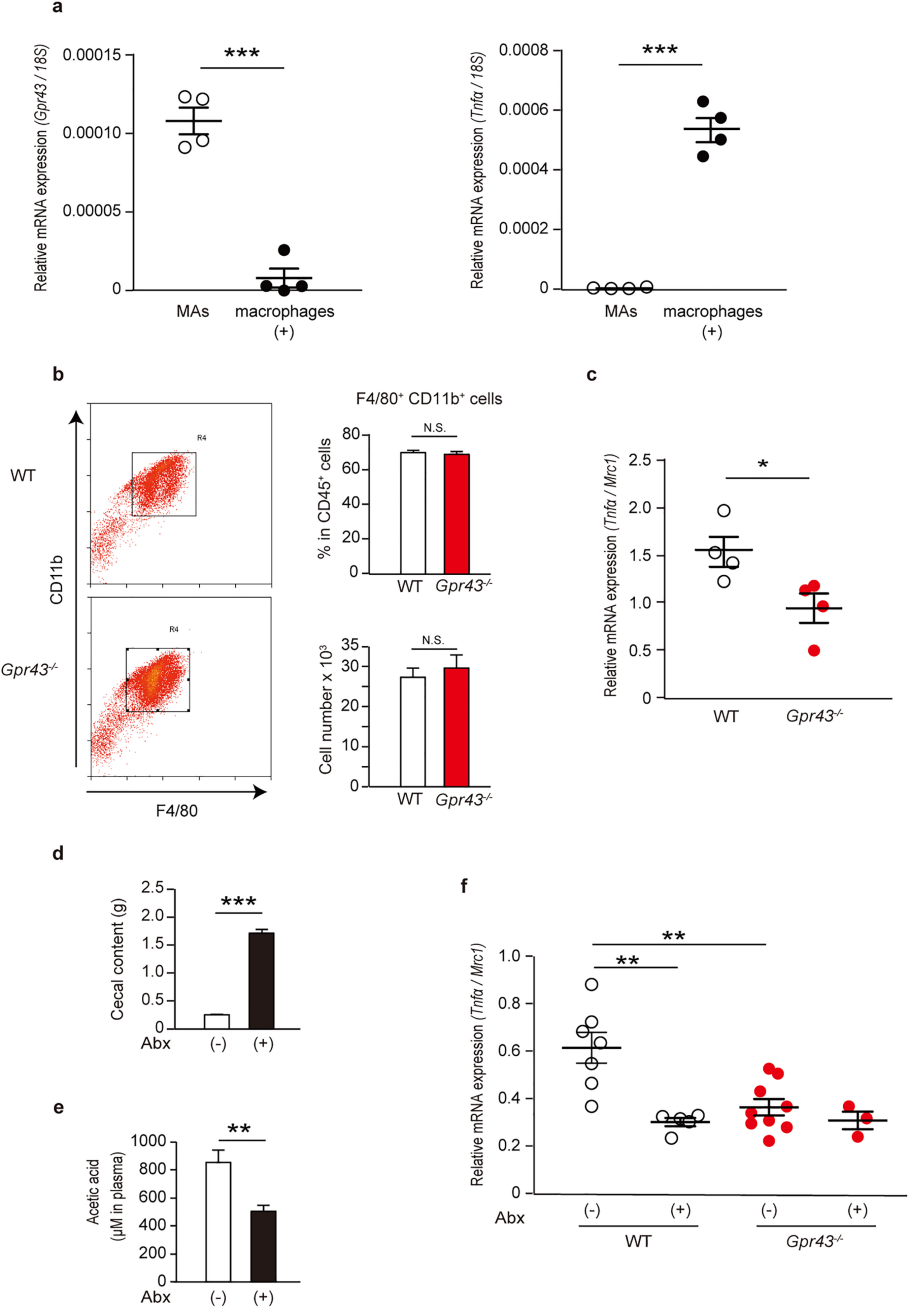
*Tnfα* mRNA is induced in adipose tissue M2-type macrophages depending on GPR43 and SCFA stimulation. (a) *Gpr43* (left) and *Tnfα* (right) mRNA expression in epididymal adipose tissue MAs and macrophage subsets of WT mice fed an NC, measured using qRT-PCR (n = 4). *18S* mRNA expression was used as an internal control. Mice were analyzed at 7−9 weeks of age. (b) Representative flow cytometric plots (left) and bar graphs summarizing the frequency and number of CD11b^+^F4/80^+^ cells by analyzing live CD45^+^ cells (right) extracted from the epididymal adipose tissue of WT and *Gpr43*^*-/-*^ mice (n = 4). Mice were analyzed at 7−8 weeks of age. (c) *Tnfα* mRNA expression in epididymal adipose tissue derived-M2-type macrophages of WT and *Gpr43*^*-/-*^ mice fed an NC, measured using qRT-PCR (n = 4). *Mrc1* mRNA expression was used as an internal control to evaluate the *Tnfα* mRNA induction from a single M2-type macrophage. Mice were analyzed at 7−8 weeks of age. (d) Cecal content weights of nontreated and antibiotics-treated WT mice fed an NC (n = 5−7). Mice were analyzed at 7–10 weeks of age. (e) Quantification of short chain fatty acids in the serum of nontreated and antibiotics-treated WT mice fed an NC (n = 5−7). Mice were analyzed at 7−10 weeks of age. (f) *Tnfα* mRNA expression in the epididymal adipose tissue derived-M2-type macrophages of nontreated and antibiotics-treated WT and *Gpr43*^*-/-*^ mice fed an NC, measured by qRT-PCR (n = 3−9). *Mrc1* mRNA expression was used as an internal control. Mice were analyzed at 7−10 weeks of age. All data are presented as mean ± S.E.M. *p < 0.05, **p < 0.01, ***p < 0.001, N.S.: not significant.

To investigate the role of GPR43 in M2-type macrophages *in vivo*, we divided epididymal adipose tissue from NC-fed WT mice into MAs and macrophage subsets and examined the expression pattern of *Gpr43* and *Tnfα* mRNA in each fraction. The *Gpr43* mRNA expression level in adipose tissue macrophages was lower than that in MAs, whereas *Tnfα* was exclusively induced in the macrophages; the induction level of *Tnfα* in macrophages was more than 100 times higher than that in MAs (Fig 6A). This result indicated the possibility that GPR43 expressed in adipose tissue macrophages regulates TNF-α signaling. To assess this, we compared the induction of *Tnfα* mRNA expression in adipose tissue M2-type macrophages from NC-fed young WT and from *Gpr43*^*-/-*^ mice. We used mice with approximately the same body weight and WAT weight (S3C and S3D Fig) because progression of obesity altered immune cell composition in the WAT and promoted inflammatory responses. Although the frequency and numbers of macrophages in adipose tissue immune cells was unperturbed in the absence of GPR43, *Tnfα* mRNA induction in M2-type macrophages was greater in WT mice than in *Gpr43*^*-/-*^ mice (Fig 6B and 6C).

Given that SCFA is a key factor in GPR43 activation, we examined whether SCFA promoted *Tnfα* induction in M2-type macrophages via GPR43 *in vivo*. To test this, WT and *Gpr43*^*-/-*^ mice were treated with antibiotics for 3 weeks and then *Tnfα* mRNA expression in adipose tissue M2-type macrophages was compared. As expected, antibiotic treatment caused cecal enlargement and significantly reduced the most abundant plasma SCFA, acetate concentrations in WT mice (Fig 6D–6E). On examination of inflammatory responses in adipose tissue M2-type macrophages, alteration of *Tnfα* mRNA expression was not seen in *Gpr43*^*-/-*^ mice regardless of antibiotic treatment. However, in WT mice, antibiotic treatment caused robust reduction of *Tnfα* mRNA expression; the expression level decreased such that it was equal to that in *Gpr43*^*-/-*^ mice (Fig 6F). Taken together, these results show that GPR43 regulates the inflammatory responses in adipose tissue M2-type macrophages depending on SCFA stimulation.

## Discussion

Obesity-associated tissue inflammation is now recognized as an important cause of decreased insulin sensitivity[33,34]. Approximately 25 years earlier, Hotamisligi *et al*. found that TNF-α production was upregulated in obese mice and that neutralization of TNF-α ameliorated insulin resistance[35]. Additionally, mice lacking TNF-α showed improved insulin sensitivity in high fat diet-induced obesity[36]; subsequent research revealed that pro-inflammatory M1-type macrophages accumulated in the adipose tissue of obese mice and that these cells were dominant sources of TNF-α for promoting insulin resistance[37]. In contrast to the concept that inflammatory signaling exerts a fundamentally negative effect on metabolism, TNF-α signaling is also recognized to be involved in homeostasis in adipose tissue and in preventing fat accumulation by inhibiting insulin signaling[38–40]. Two recent studies established the role of TNF-α signaling in adipose tissue[26,41]. Asterholm *et al*. showed that local TNF-α signaling derived from the adipose tissue was in fact required for proper adipose tissue remodeling and healthy expansion[41]. Bapat *et al*. also observed that blocking inflammatory pathways in aging adipose tissue resulted in impairment of insulin sensitivity[26]. Taken together, these results provide evidence that distinct immune responses orchestrate unique features of steady state- and obesity-associated metabolic regulation. In steady states, maintaining a certain degree of inflammation by M2-type macrophages or other types of immune cells is beneficial for remodeling of adipose tissue and its metabolic function; however, increased inflammation of M1-type macrophages in obesity can lead to detrimental metabolic consequences.

Our current data support that SCFA-activated GPR43 played distinct functions in M1- and M2-type macrophages. Although more in-depth information is required regarding the underlying mechanism, we propose that GPR43 promotes *Tnfα* mRNA induction in M2-type macrophages, depending on SCFA stimulation, and supports increase in metabolic activity by impeding fat accumulation. The findings related to this were consistent with those of Bapat *et al*[26]. The role of GPR43 in M1-type macrophages *in vivo* has not yet been clarified. It is possible that GPR43 suppresses the inflammatory responses in adipose tissue M1-type macrophages and prevents the progression of obesity, because *Tnfα* mRNA induction from thioglycollate-elicited PECs stimulated with SCFA was suppressed depending on GPR43 (S3B Fig). Moreover, we observed that *Gpr43* mRNA expression slightly increased in the mesenteric adipose tissue SVF with progression of obesity *in vivo* and was also higher in LPS-stimulated Raw264.7 cells (mouse macrophage cell line) than in non-treated Raw264.7 cells *in vitro* (S1D and S3E Figs). To obtain more in-depth information regarding the functions of GPR43 in macrophages, further investigation involving macrophage-specific conditional *Gpr43*^*-/*^ mice is required.

The mechanism underlying the difference between M1- and M2-type macrophages with respect to different GPR43-mediated inflammatory responses is not known. GPR43 couples to either G_i/o_ or G_q_ subunits, and we hypothesized that GPR43 signaling occurred via divergent G protein pathways that can selectively promote or inhibit inflammatory responses in immune cells. Indeed, a recent study investigating glucose-stimulated insulin secretion (GSIS) in murine islets revealed that selective GPR43 signaling through either G_i/o_ or G_q_ translated into disparate effects on GSIS[42]. Since GPR43 signaling is likely to be even more complex, further investigation will be required to clarify the mechanism underlying GPR43-mediated inflammatory responses.

Recent studies have implicated involvement of GPR43 in chronic inflammatory diseases such as obesity, colitis, and cancer[18,22,43]. However, whether GPR43 prevents or promotes inflammation is inconsistent between studies. This demonstrates the importance of cell types and their location when interpreting downstream mechanisms of GPR43 signaling. Indeed, our results showed that GPR43 played distinct functions *in vitro* depending on the types of immune cells. Furthermore, in *aP2-Gpr43TG* mice, high levels of TNF-α production were seen only in the WAT but not in the liver and muscle, indicating that the situation is more complex *in vivo*. For instance, recent findings suggest that (a) adipocytes collaborate with certain immune cells and directly regulate the activation and proliferation of adipose immune cells by secreting various adipokines such as adiponectin and MCP-1 [44,45] and that (b) adipocytes also act as antigen presenting cells to immune cells in adipose tissue inflammation[46]. Thus, since the crosstalk between tissue-specific cells such as adipocytes and various types of immune cells may affect the biological and molecular functions of GPR43, further information is required on the cell-type-specific and tissue-specific functions of GPR43 for elucidation of all aspects of involvement of GPR43 in inflammatory responses.

In conclusion, the findings of our previous study[18] and our current study suggest that GPR43 plays an important role in suppressing fat accumulation by impairing insulin signaling directly via the PLC-PKC-PTEN pathway and indirectly via TNF-α production by M2-type macrophages, because previous studies established that TNF-α signaling of adipocytes led to decreased expression of the insulin-responsive glucose transporter GLUT4 in adipose tissue and impaired fat accumulation [38–40]. Our finding indicates a new example of the interconnectedness between adipocytes and immune cells in steady-state adipose tissue. Dissecting the complex interactions that occur between immune and metabolic systems via GPR43 will provide important insights into possible therapeutic strategies for treating obesity and associated diseases.

## Materials and methods

### Animals

C57BL6/J mice were housed under a 12-h light–dark cycle and given regular chow (MF, Oriental Yeast Co). All experimental procedures involving mice were performed according to protocols approved by the Committee on the Ethics of Animal Experiments of the Tokyo University of Agriculture and Technology. (Permit Number: 28–87). For HFD studies, 4-week-old male mice were placed on a D12492 diet (60% kcal fat, Research Diets) for 12 weeks. The generation of *Gpr43*^*-/-*^ was described previously[18]; the mice were maintained on a C57BL6/J genetic background. *aP2-Gpr43TG* mice were generated as previously[18]. For antibiotic treatment, 4-6-week-old mice were treated with ampicillin (Nacalai Tesque; 0.4 mg/ml), neomycin (Nacalai Tesque; 0.4 mg/ml), metronidazole (Wako; 0.4 mg/ml), gentamicin (Sigma; 0.4 mg/ml) and vancomycin (Sigma; 0.2 mg/ml) in drinking water for 3 weeks. For collection of blood and tissue samples, mice were sacrificed by anesthesia with somnopentyl. All efforts were made to minimize suffering.

### RNA extraction and real-time quantitative RT-PCR

Total RNA was extracted using an RNeasy Mini Kit (Qiagen) and subjected to DNase treatment (Invitrogen), and reverse transcription were performed using Moloney murine leukemia virus reverse transcriptase (Invitrogen). Quantitative reverse transcriptase PCR (qRT-PCR) analysis was performed with using SYBR *Premix Ex Taq* II (TAKARA) and the ABI7300 apparatus (Applied Biosystems). Primer sequences for *Gpr43* (mouse), *Tnfα* (mouse) and *GPR43* (human) have been described previously[18]. Other primer sequences are as follows: *F4/80* (mouse), 5’- GATGTGGAGGATGGGAGATG -3’ (forward) and 5’- ACAGCAGGAAGGTGGCTATG -3’ (reverse); *Mcp-1* (mouse), 5’- AATCTGAAGCTAATGCATCC -3’ (forward) and 5’- GTGTTGAATCTGGATTCACA -3’ (reverse); *Il6* (mouse), 5’- GGAGTACCATAGCTACCTGG -3’ (forward) and 5’- AGGAATGTCCACAAACTGAT -3’ (reverse); *Inos* (mouse), 5’- TGGTGGTGACAAGCACATTT -3’ (forward) and 5’- AAGGCCAAACACAGCATACC -3’ (reverse); *Mrc1* (mouse), 5’- CAAGGAAGGTTGGCATTTGT -3’ (forward) and 5’- CCTTTCAGTCCTTTGCAAGC -3’ (reverse); *18S* (mouse), 5’- ACGCTGAGCCAGTCAGTGTA -3’ (forward) and 5’- CTTAGAGGGACAAGTGGCG -3’ (reverse), *Pparg2* (mouse), 5’- GCTGTTATGGGTGAAACTCTGG -3’ (forward) and 5’- TTCTTGTGAAGTGCTCATAGGC -3’ (reverse), *Cd45* (mouse), 5’- tcgtgcccaaacaaattaca -3’ (forward) and 5’- taggcttaggcgtttctgga -3’ (reverse).

### Enzyme-linked immunosorbent assay (ELISA)

Murine TNF-α was measured by ELISA. TNF-α ELISA kits was obtained from R&D systems.

### Isolation of adipose tissue MAs and SVF

Epididymal, mesenteric and subcutaneous adipose depots were dissected from mice and minced in sterile PBS. The tissues were then digested with 1 mg/ml collagenase type I (Sigma) in PBS at 37°C for 30 min with gentle shaking. The suspension was then passed through a 100-μm mesh to remove undigested clumps and debris. The flow-through was allowed to centrifuge at 300rpm for 3 min to separate the floating MAs fraction. After removing MAs fraction, the SVF was collected by centrifugation at 1500rpm for 10 min. The resultant isolated cells were subjected to FACS analysis.

### Flow cytometry

The pellet containing the SVF was washed once with ice-cold FACS buffer (2% FCS in PBS). After washing, the cells were stained with phycoerythrin (PE)-Cy7 conjugated anti-CD45 (BioLegend), allophycocyanin (APC) conjugated anti-CD11b (BioLegend) and biotin conjugated anti-F4/80 (BioLegend) following by incubation with Alexa Fluor 488 conjugated streptavidin (Invitrogen) as secondary antibody. Macrophage subsets (CD45^+^CD11b^+^F4/80^+^ cells) and non-macrophages immune cells (CD45^+^CD11b^-^F4/80^-^ cells) were each sorted by a MoFo XDP (BECMAN COULTER).

### GTT and ITTs

For GTT, 24-h-fasted mice were given 1.5 mg of glucose (Wako) per gram of body weight (1.5 mg / g) intraperitoneally. For ITT, 3-h-fasted mice were given human insulin (0.75 mU / g, Sigma) intraperitoneally. Blood glucose concentration was monitored using a OneTouch Ultra before injection and at 15, 30, 60, 90 and 120 min after injection.

### Western blotting

M2 polarized bone marrow derived macrophages were cultured in serum-free RPMI 1640 (Gibco) for 3 h, and then stimulated with acetate (10mM;Wako). After 10 min, cells were lysed in TNE buffer and centrifuged at 14,000 g for 20 min at 4°C. The supernatants were resolved by SDS gel electrophoresis and blotted onto a nitrocellulose membrane. Primary antibodies used were follows: ERK1/2 (Cell signaling; rabbit, 1:1,000) and phosphorylated ERK1/2 (Cell signaling; rabbit, 1:1,000). The secondary antibody used was a horseradish peroxidase-conjugated goat anti-rabbit antibody (GE Healthcare; 1:2000). Immunoreactive bands were visualized using an enhanced chemiluminescence detection system. Image J (National Institutes of Health) was used to quantify the integrated density of each band.

### Immunofluorescence

Adipose tissue was fixed in 10% Formalin Neutral Buffer Solution (Wako). Fixed sections were embedded in paraffin, sectioned and stained with the following primary antibodies: F4/80 (Abcam; rat, 1:1,000) and Caveolin1 (BD Biosciences; mouse, 1:200). The secondary antibody used was a Alexa Fluor 488-conjugated anti-rat antibody (Invitrogen; 1:200) or Alexa Fluor 555-conjugated anti-mouse antibody (Invitrogen; 1:200), respectively. At the end of the staining, slides were washed and incubated with DAPI (Sigma) and mounted with Fluoromount-G (Southern Biotech). Fluorescence images were obtained with a fluorescence microscopy LSM710 (Carl Zeiss).

### Bone marrow derived macrophages (BMDM)

The bone marrow cells from mice were isolated and incubated in RPMI 1640 (Gibco) supplemented with mouse macrophage colony-stimulating factor mouse (MCSF 50 ng/mL; BioLegend) and 10% fetal bovine serum (FBS). The medium was replaced with fresh RPMI 1640 medium containing with MCSF (50 ng/mL) and 10% FBS every two days. On day 6 in culture, cells were activated (M1 condition) with MCSF (50 ng/mL), LPS (10 ng/mL; SIGMA) and IFN-γ (100 ng/mL; BioLegend), alternatively activated (M2 condition) with MCSF (50 ng/mL) and IL-4 (20ng/mL; BioLegend) for 24 h. After polarization, cells were stimulated with acetate (10 mM) for 7 h.

### Thioglycollate- elicited peritoneal exudate cells

Mice were euthanized 72 h after intraperitoneally injection of 3 ml 4% thioglycollate. Peritoneal cells were harvested by injecting 7 mL of FACS buffer (2% FCS in PBS) into PerC. Macrophages were enriched by adherence by plating peritoneal cells in RPMI 1640 medium for 3 h. Non-adherent cells were removed, then treated with serum-free for 12 h later and stimulated with acetate (10 mM) for 7 h.

### Culture of Raw264.7 cells

Mouse macrophage cell line (Raw264.7 cells) were cultured with Dulbecco’s modified Eagle’s medium (DMEM; Sigma) containing 1% penicillin–streptomycin solution (Gibco) and 10% FBS. Raw cells were stimulated with LPS (100 ng/mL; SIGMA) for 24 h and then harvested to isolate RNA.

### SCFA analyses by GC-MS

Sample preparation protocol for SCFA determination in serum modified the method described previously^18^. Then, collected and pooled ether layers were transferred to glass vials for GC-MS analysis. Analyses were performed with a GCMS-QP2010 Ultra (Shimadzu) as described previously^18^.

### Statistical analysis

Values are presented as the mean ± s.e.m. Differences between groups were examined for statistical significance using Student’s t-test (two groups) or one-way analysis of variance followed by Tukey’s multiple comparison test. P-values < 0.05 were considered statistically significant.

## Supporting information

S1 Fig *Gpr43* is expressed in the adipose tissue SVF of HFD-fed mice. (a−c) Expression of *Pparg2* (the marker of mature adipocytes) and *Cd45* (the marker of leucocytes) mRNA in the adipose tissue MAs and SVF of NC-fed mice by qRT-PCR (n = 3). Mesenteric adipose tissue (a), epididymal adipose tissue (b) and subcutaneous adipose tissue (c). (d−f) Expression of *Gpr43* mRNA in the adipose tissue SVF of HFD-fed mice by qRT-PCR (n = 3). Mesenteric adipose tissue (d), epididymal adipose tissue (e) and subcutaneous adipose tissue (f). *18S* mRNA expression was used as an internal control. All data are presented as mean ± S.E.M. *p < 0.05, **p < 0.01, ***p < 0.001, N.S.: not significant. SVF: stromal vascular fraction.
(TIF)

S2 Fig Sorting strategies used to generate adipose tissue macrophages. Adipose tissue macrophages were generated using a cocktail of several antibodies. Adipose tissue macrophage subsets were sorted by gating on CD45^+^CD11b^+^F4/80^+^ cells. Adipose tissue non-macrophage immune cells were sorted by gating on CD45^+^CD11b^-^F4/80^-^ cells. We confirmed that the purity of the sorted cells was more than 93%.
(TIF)

S3 Fig GPR43 plays distinct functions according to the macrophages types. (a) Effects of acetate (10 mM) on ERK1/2 phosphorylation in WT and *Gpr43*^*-/-*^ M2-type bone marrow-derived macrophages. Cells were cultured for 3 h in serum-free medium and stimulated with acetate (10 mM) for 10 min. (b) Quantitative RT-PCR analysis of *Tnfα* mRNA expression in WT and *Gpr43*^*-/-*^ thioglycollate-elicited PECs stimulated for 7 h with acetate (10 mM) (n = 3). *18S* mRNA expression was used as an internal control. Body weights (c) and tissue weights (d) of WT and *Gpr43*^*-/-*^ mice used in the analysis shown in Fig 6C and 6D (n = 4). Mice were analyzed at 7−8 weeks of age. (e) *Gpr43* mRNA expression in LPS-treated (100 ng/mL, 24 h) or nontreated Raw264.7 cells. *18S* mRNA expression was used as an internal control. All data are presented as mean ± S.E.M. *p < 0.05, **p < 0.01, ***p < 0.001, N.S.; not significant. Data are representative of two (a) or three (c) experiments.
(TIF)
