# Supplementary figures and images for: The short chain fatty acid receptor GPR43 regulates inflammatory signals in adipose tissue M2-type macrophages
Source: PLoS One. 2017 Jul 10;12(7):e0179696. doi: 10.1371/journal.pone.0179696 (PMC5503175; doi:10.1371/journal.pone.0179696)

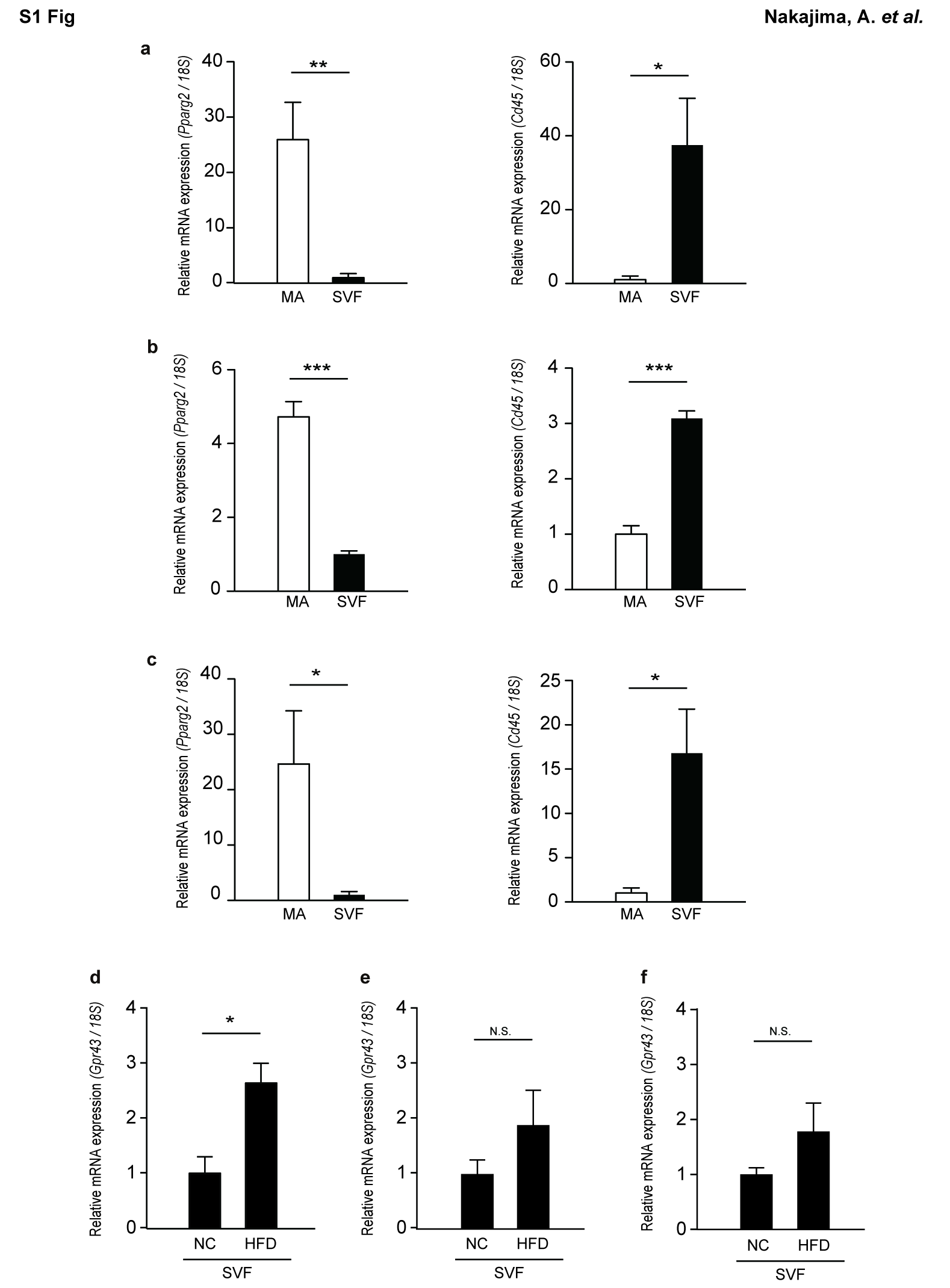

Supplement: S1 Fig — (a−c) Expression of Pparg2 (the marker of mature adipocytes) and Cd45 (the marker of leucocytes) mRNA in the adipose tissue MAs and SVF of NC-fed mice by qRT-PCR (n = 3). Mesenteric adipose tissue (a), epididymal adipose tissue (b) and subcutaneous adipose tissue (c). (d−f) Expression of Gpr43 mRNA in the adipose tissue SVF of HFD-fed mice by qRT-PCR (n = 3). Mesenteric adipose tissue (d), epididymal adipose tissue (e) and subcutaneous adipose tissue (f). 18S mRNA expression was used as an internal control. All data are presented as mean ± S.E.M. *p < 0.05, **p < 0.01, ***p < 0.001, N.S.: not significant. SVF: stromal vascular fraction. (TIF) [file pone.0179696.s001.tif]

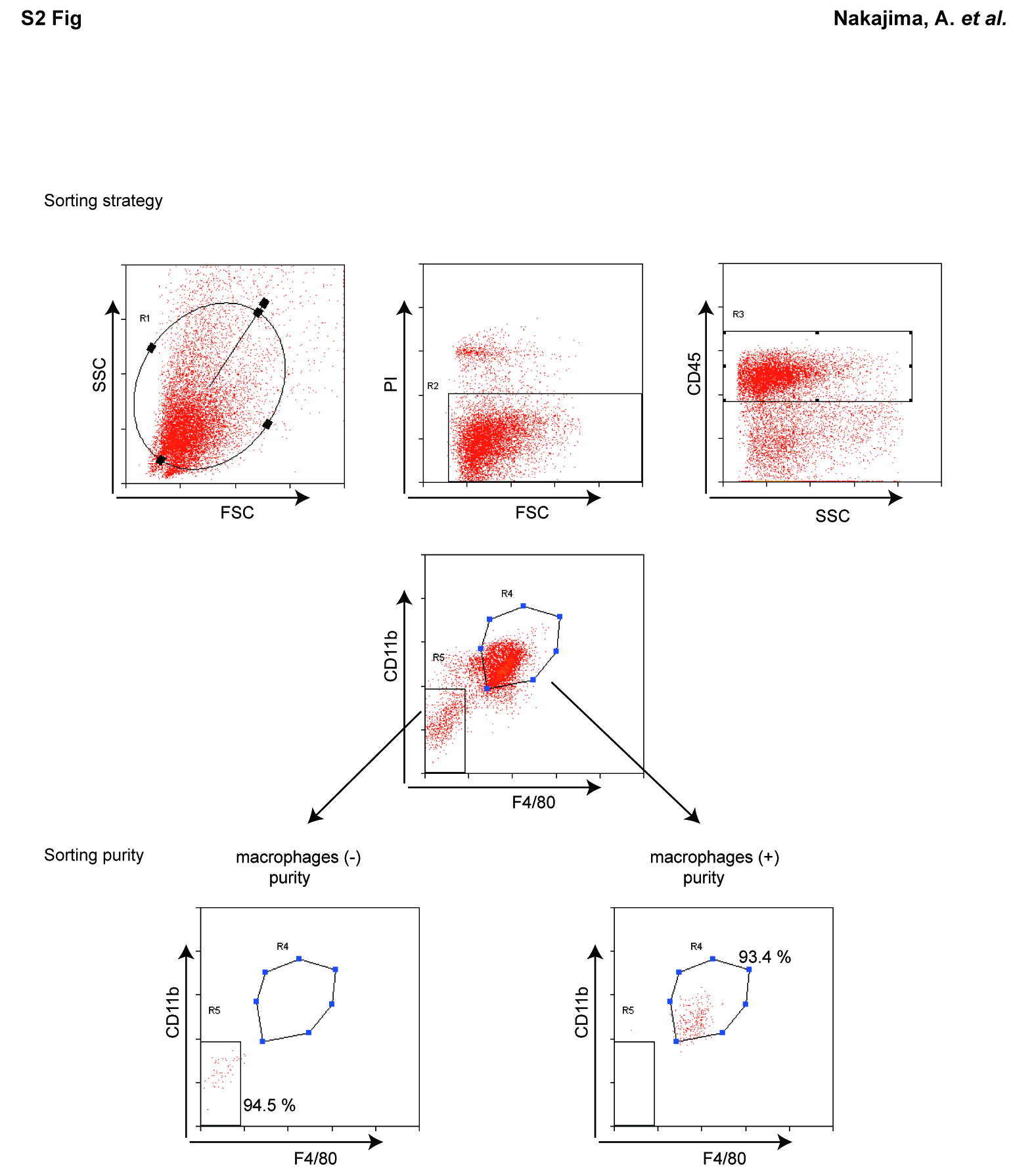

Supplement: S2 Fig — Adipose tissue macrophages were generated using a cocktail of several antibodies. Adipose tissue macrophage subsets were sorted by gating on CD45+CD11b+F4/80+ cells. Adipose tissue non-macrophage immune cells were sorted by gating on CD45+CD11b-F4/80- cells. We confirmed that the purity of the sorted cells was more than 93%. (TIF) [file pone.0179696.s002.tif]

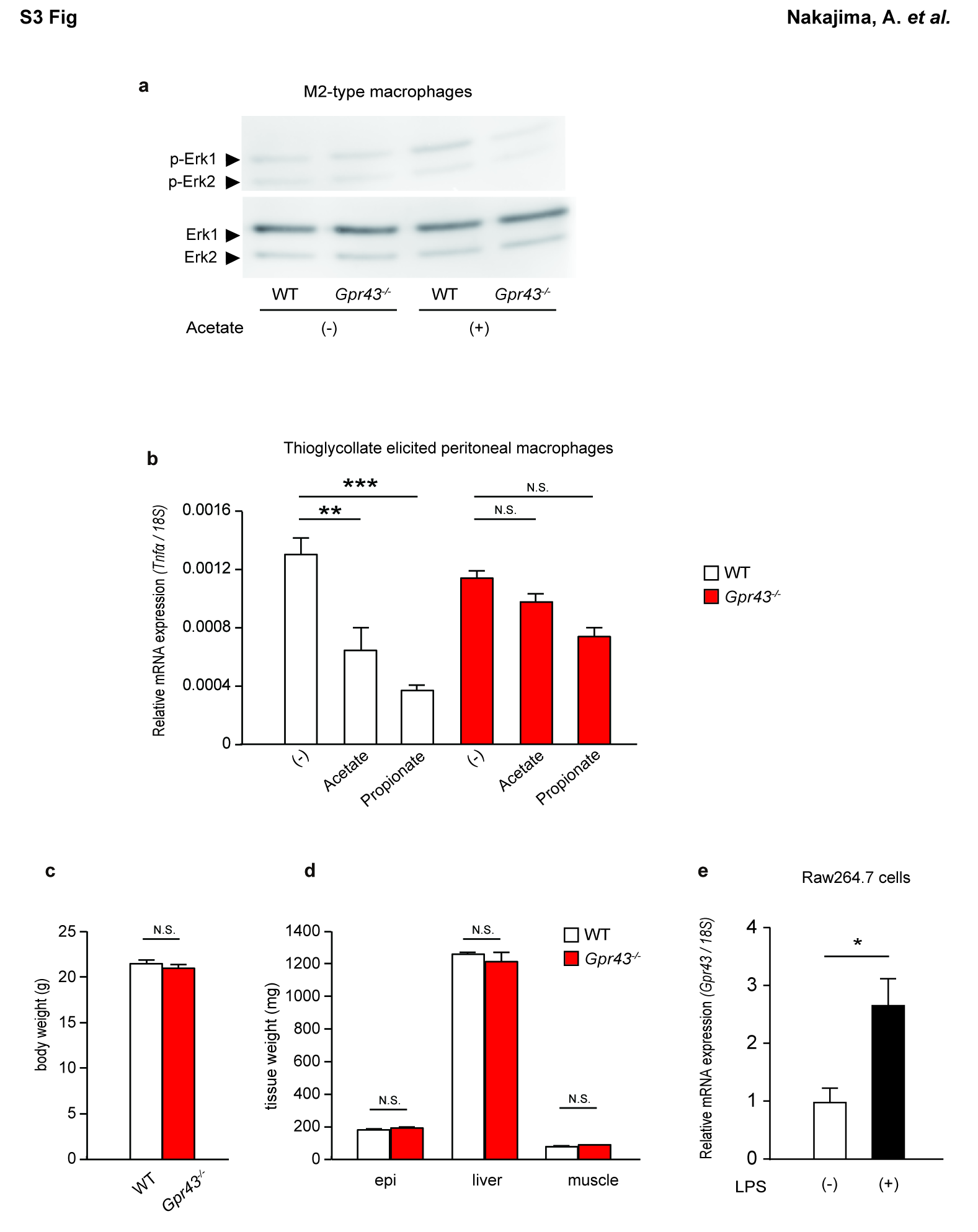

Supplement: S3 Fig — (a) Effects of acetate (10 mM) on ERK1/2 phosphorylation in WT and Gpr43-/- M2-type bone marrow-derived macrophages. Cells were cultured for 3 h in serum-free medium and stimulated with acetate (10 mM) for 10 min. (b) Quantitative RT-PCR analysis of Tnfα mRNA expression in WT and Gpr43-/- thioglycollate-elicited PECs stimulated for 7 h with acetate (10 mM) (n = 3). 18S mRNA expression was used as an internal control. Body weights (c) and tissue weights (d) of WT and Gpr43-/- mice used in the analysis shown in Fig 6C and 6D (n = 4). Mice were analyzed at 7−8 weeks of age. (e) Gpr43 mRNA expression in LPS-treated (100 ng/mL, 24 h) or nontreated Raw264.7 cells. 18S mRNA expression was used as an internal control. All data are presented as mean ± S.E.M. *p < 0.05, **p < 0.01, ***p < 0.001, N.S.; not significant. Data are representative of two (a) or three (c) experiments. (TIF) [file pone.0179696.s003.tif]
